# Evidence for a Pathogenic Role of Different Mutations at Codon 188 of *PRNP*

**DOI:** 10.1371/journal.pone.0002147

**Published:** 2008-05-14

**Authors:** Sigrun Roeber, Eva-Maria Grasbon-Frodl, Otto Windl, Bjarne Krebs, Wei Xiang, Caren Vollmert, Thomas Illig, Andreas Schröter, Thomas Arzberger, Petra Weber, Inga Zerr, Hans A. Kretzschmar

**Affiliations:** 1 Center for Neuropathology and Prion Research, Ludwig-Maximilians-University, München, Germany; 2 Institute for Epidemiology, Forschungszentrum für Umwelt und Gesundheit (GSF) München, Neuherberg, Germany; 3 Department of Neurology, Georg-August-University, Göttingen, Germany; University of Giessen, Germany

## Abstract

Clinical and pathological changes in familial Creutzfeldt-Jakob disease (CJD) cases may be similar or indistinguishable from sporadic CJD. Therefore determination of novel mutations in *PRNP* remains of major importance.

We identified two different rare mutations in codon 188 of the prion protein gene (*PRNP)* in four patients suffering from a disease clinically very similar to the major subtype of sporadic CJD. Both mutations result in an exchange of the amino acid residue threonine for a highly basic residue, either arginine (T188R) or lysine (T188K). The T188R mutation was found in one patient and the T188K mutation in three patients. The prevalence of mutations at codon 188 of *PRNP* was tested in 593 sporadic CJD cases and 735 healthy individuals. Neither mutation was found. The data presented here argue in favor of T188K being a pathogenic mutation causing genetic CJD. Since one individual with this mutation, who is the father of a clinically affected patient with T188K mutation, is now 79 years old and shows no signs of disease, this mutation is likely associated with a penetrance under 100%. Further observations will have to show whether T188R is a pathogenic mutation.

## Introduction

Human prion diseases can be idiopathic, acquired or genetic. The majority of cases are idiopathic and are generally referred to as sporadic Creutzfeldt-Jakob disease (sCJD). About 10%–15% of human prion diseases are inherited in an autosomal, dominant fashion and in all cases a mutation in the coding region of the human prion protein gene (*PRNP*) has been found [1], [2]. Depending on their clinical and pathological phenotypes, the inherited prion diseases have been termed as genetic (familial) CJD, Gerstmann-Sträussler-Scheinker syndrome (GSS) or fatal familial insomnia (FFI). More than 20 different point mutations are known to date which are found to be associated or genetically linked with inherited prion diseases. These point mutations cluster in the central and in the more C-terminal part of the prion protein (PrP) encoded by *PRNP*.

The most common mutations in inherited human prion diseases are well described and their phenotypic range has been determined. The glutamate to lysine mutation at codon 200 (E200K) is causative of the majority of familial CJD cases, the proline to leucine mutation at codon 102 (P102L) for GSS and the aspartate to asparagine mutation at codon 178 (D178N) coupled with methionine at codon 129 for FFI [3]–[7]. Whereas the clinical and histopathological spectrum of GSS and FFI differ from sCJD, the spectrum of familial CJD overlaps in many cases with the spectrum of sCJD [4] . In the absence of genetic testing of suspected sCJD, mutations of *PRNP* often would not be detected and familial CJD would be underreported. The determination of novel mutations in *PRNP* remains of major importance for the following reasons. (1) The identification of new mutations might identify some dementing or neurodegenerative clinical syndromes as prion diseases, thus expanding the spectrum of prion diseases and improving the differential diagnosis during lifetime. (2) Every novel mutation adds valuable insights to our understanding as to how such changes in PrP cause prion diseases. (3) Several mutations giving rise to a phenotype mimicking sCJD may have been overlooked due to an examination bias towards known mutations. This may lead to erroneous diagnoses with implications for the affected families.

Here we report two different rare mutations at codon 188 of *PRNP* in four cases of clinically diagnosed and one autopsy proven CJD case and argue that mutations at this position are causative of genetic CJD. The mutations are located in a highly conserved region and alter the codon 188 from threonine (T) to a basic amino acid residue, either arginine (T188R) or lysine (T188K).

## Materials and Methods

### Clinical history/Neuropathology

Patients A–D were identified as part of the German CJD surveillance program, where about 2000 patients were investigated clinically, genetically and many of them neuropathologically [5], [8]. Patients A–C were not autopsied. For patient D informed consent for autopsy was received from parents. The relatives of patient D (sister (E), father (F), mother (G)) were clinically inconspicuous and received predictive genetic analysis after signed informed consent.

One year before the death of patient D we received a 3-mm tissue sample from a brain biopsy, after her death a brain autopsy was performed. The left hemisphere was fixed in formalin and the right hemisphere was frozen (−80°C). Histological examination was performed on 4 µm thick sections of 17 regions of formalin-fixed and paraffin embedded cerebrum, cerebellum and brainstem. Hematoxylin and eosin (H&E) as well as Gallyas silver stain, Luxol fast blue–periodic acid Schiff (LFB-PAS) and Bielschowsky stains were performed using standard techniques. Immunohistochemistry was performed with antibodies directed against PrP (L42, 1∶100, gift from M. Groschup), phosphorylated tau (AT8, 1∶200, Innogenetics, Ghent, Belgium), α-synuclein (15G7, 1∶10, own production, [9]) and αB-crystalline (1∶500, Calbiochem, Nottingham, UK). Paraffin-embedded tissue (PET) blotting was performed using the antibody 12F10 (gift from J. Grassi) as described elsewhere [10].

### Genetic analysis

For genetic analysis DNA was extracted from blood lymphocytes or from frozen post-mortem brain tissue according to standard procedures. The coding region of *PRNP* was amplified by the polymerase chain reaction (PCR) and first subjected to single-stranded conformational polymorphism (SSCP) analysis. In case a mutation was indicated, the complete open reading frame of *PRNP* was analyzed by direct sequencing of PCR products using the automated LICOR 4200 DNA analyzer as described previously [5].

The mutation in patient A was detected by the procedure as described. The mutations in patients B–D and persons E–G were detected by direct sequencing without prior SSCP analysis. In patient C a silent mutation at codon 117 complicated the analysis due to a polymorphism co-migrating in the region of the 5′-primer leading to preferential amplification of the allele with the silent mutation [11]. The re-analysis of the coding region of *PRNP* by PCR was then performed with a different 5′-primer [5].

The amino acid at codon 129 in coupling with the mutation was determined by cloning. For this purpose, the PCR product was cleaved with *Eco*RI (part of primer B [12]) and *Xma*I (at codon 40 of the prion protein), the large fragment was purified and cloned into the appropriate cleavage sites of pBluescriptIIKS(-) (Stratagene, La Jolla, CA). The mutation and the respective codon 129 were verified by sequencing of three independent clones.

### Single nucleotide polymorphism (SNP) analysis

The prevalence of mutations at codon 188 of *PRNP* was tested in a sCJD cohort (593 sCJD cases; 387 “definite” and 206 “probable” sCJD), received from a large German study on the genetics of prion diseases, and 735 healthy individuals [13]. The latter were taken from a population-based German study performed in the city and region of Augsburg (KORA; Kooperative Gesundheitsforschung im Raum Augsburg, Survey 4, 2000), which is a representative sample of the adult general population of German nationality and were matched for age and gender [14], [15]. All study participants gave informed written consent according to the Bavarian Ethics committee and the Ethics committee of the Ludwig-Maximilians-University of Munich.

Genomic DNA of sCJD patients was extracted from blood lymphocytes according to standard procedures. The SNP analysis for both mutations (T188R, T188K) was performed using matrix assisted laser desorption/ionisation time of flight mass spectrometry (MALDI-TOF MS, Mass Array™, Sequenom, San Diego, CA, USA). Briefly, genomic DNA of each subject was tested individually using PCR with primers 3′-ACGTTGGATGTCATCTTAACGTCGGTCTCG-5′ and 3′-ACGTTGGATGCACGACTGCGTCAATATCAC-5′ generating products of 108 bp. Following purification of the PCR products homogenous MassEXTEND reactions (hME™, Sequenom, San Diego, CA, USA) were performed by adding a mixture of dideoxy and deoxynucleotides and the test-specific MassEXTEND primer 3′-TTGGTGGTTGTGGTGACC-5′, which is complementary to the template at a region directly adjacent to the polymorphic site. The hME products were spotted onto a 384 SpectroCHIP microarray for fully automated mass spectrometry analysis (Biflex mass spectrometer, Bruker Daltonik GmbH, Bremen, Germany; MassArray™, Sequenom, San Diego, CA, USA).

### Western Blot

For Western blot analysis tissue from the frontal cortex of patient D was homogenised in 9 volumes (wt/vol) of lysis buffer (0.5% Nonidet P-40, 0.5% DOC, 10 mM EDTA, 100 mM NaCl, 100 mM Tris, pH 6.9 at 37°C). After PK digestion (100 µg/ml, 1h at 37°C) cleared homogenates were subjected to SDS-PAGE (12% NuPage, Invitrogen, Karlsruhe, Germany) and blotted on PVDF-membranes [16]. For comparison we used brain homogenates from two sCJD cases (MM1 and VV2). The monoclonal anti-PrP antibody 3F4 (1∶3000, DAKO), recognizing the epitope 109–112 of PrP, was used for immunodetection. Protease-resistant PrP was visualized by enhanced chemiluminescence reaction (GE Healthcare, Freiburg, Germany).

## Results

### Clinical history

#### Patient A

The patient was of Caucasian descent, born and raised in Germany. She spent the last 33 years of her life in Indonesia. At age 66 she presented with progressive visual impairment followed several months later by progressive dementia and slight ataxia. Her medical history was unremarkable aside from a malaria episode. On examination during a visit in Germany, 12 months after onset of the disease, the patient was oriented towards her person, but disoriented in time. She had impaired short-term memory and reported increased exhaustion, tiredness and weight loss. Examination of her CSF revealed neuron-specific enolase (NSE) of 38 ng/ml (cut-off 35 ng/ml for an optimum sensitivity and specificity for the diagnosis of CJD [17]), intrathecal IgG-production and 14-3-3 proteins. In the cerebral magnetic resonance imaging (MRI) 12 months after onset only one small (7 mm) lesion was found in the frontal white matter on both sides. They were hyperintense on T2, invisible on T1, non-enhancing and interpreted as “myelinoclastic lesions”. The electroencephalogram (EEG) showed typical periodic sharp-wave complexes (PSWCs). The sensory evoked potentials (SEP) and acoustical evoked potentials (AEP) were normal, the monocular visual evoked potentials (VEP) indicated a demyelinating lesion on both sides, most likely localized in the prechiasmatic optic nerves. The patient died 16 months after the onset of her disease. An autopsy was not performed. A family history of dementing illnesses was not known. The patient's father had succumbed to pleuritis at a very young age (31 y).

#### Patient B

The patient was born in Germany and lived there for her whole life. At age 76 she presented with coordination problems of the left hand and slight disturbances of her gait. Involuntary movements, alien limb-sign, focal dystonia, rigor of upper extremities, apraxia and ataxia were found. In a first Mini Mental State Examination (MMSE) test three months after the onset the patient scored normal. The initial diagnosis was corticobasal degeneration. Only one month later she was severely demented. She developed visual problems, up-beat nystagmus and dysarthria. In the CSF the NSE level was 28 ng/ml (elevated, but below the cut-off of 35 ng/ml for CJD diagnosis) and the Western blot for 14-3-3 was positive. The initial MRI, two months after onset, showed moderate atrophy of the fronto-parietal cortex and little unspecific white matter lesions fronto-parietal. The MRI one month prior death revealed an abnormal T2 signal in the basal ganglia on both sides. The single-photon emission computed tomography (SPECT) revealed a hypoperfusion of the parietal cortex bilaterally. The EEG showed PSWCs. The VEP revealed a delay of the P100 component bilaterally. The patient died 5 months after the onset of the disease and was not autopsied. At that time, the younger sister (59 y) was healthy. The mother of the patient was reported having died aged 64 from a rapidly progressing dementia. Yet, the parents of the patient's mother died late in life (74 y and 89 y) of non-dementing disorders. The son of the patient died from an unknown liver disease at the age of 51 y.

#### Patient C

The patient was born and lived for her whole life in Germany within 140 kilometres from patient B. At age 69 she presented with vertigo, personality changes and visual disturbances followed soon after by involuntary movements of the left limbs and a hemiparesis of the left side. Short-term memory was impaired and myoclonus was present. Signs of ataxia were diagnosed on neurological examination three months after onset. The cerebral MRI two and three months after onset showed mild enlargement of the ventricular spaces and periventricular unspecific white matter lesions in the centrum semiovale. PSWCs were indicated in the EEG. The examination of CSF revealed 14-3-3 proteins and a clearly elevated NSE level of 140 ng/ml (cut-off 35 ng/ml for CJD diagnosis). The patient's condition deteriorated rapidly and she was bed-ridden with akinetic mutism four months after the onset of the disease. Three months later, the patient died and no autopsy was performed. No family history of a dementing disease was known, both her parents died of unrelated diseases aged 77 and 87 years.

#### Patient D

The patient lived for her whole life in Germany within 130 kilometres from patient B and 10 kilometres from patient C. One year preceding her death this 58 year old woman subacutely developed dizziness, aphasia, alexia, apraxia and dysdiadochokinesia of the right hand. The symptoms were clearly progressive during the next two weeks. The MRI, one month after onset, showed cortical signal enhancement of the left temporal lobe. The EEG showed no regular activity with intermittent bilateral PSWCs. Elevated tau protein (2218 pg/ml) and 14-3-3 proteins were detectable in the CSF. According to the criteria for clinical diagnosis of CJD the diagnosis of “probable CJD” was made [18]. During the following weeks the disease progressed to a condition of mutism, the muscle tone increased and myoclonic jerks appeared. She died one year after the onset of clinical signs in a nursing home. No similar diseases were reported in her family.

### Genetics

#### Patient A

**Figure 1:**
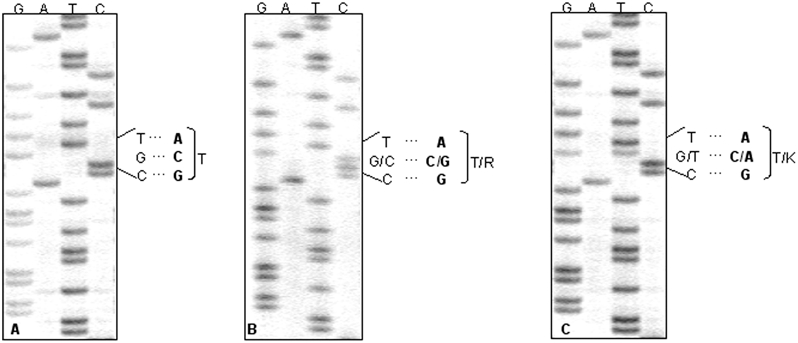
Sequence analysis of *PRNP* in three patients with prion diseases. The coding region was sequenced using fluorescence-labeled primers on an automated sequencing system (LI-COR, Lincoln, Neb.). Short fragments of *PRNP* of (A) a patient with the normal codon 188, (B) patient A with the T188R mutation and (C) patient B with the T188K mutation are shown using a primer for the sequencing that reads the antisense strand.

The analysis of the coding region of *PRNP* showed an abnormal pattern in the SSCP analysis compared to the normal *PRNP* of patients without sequence aberrations and to *PRNP* of patients with various known mutations. Sequencing revealed a cytosine to guanine transversion at the second position at codon 188 on one allele resulting in an amino acid exchange of threonine (ACG) to arginine (AGG) (T188R) (Figure 1B). The amino acid at codon 129 was valine (V) at both alleles.

#### Patients B,C,D and persons E,F,G

Samples of patients B,C,D and persons E,F,G were directly sequenced after PCR amplification. A transversion to adenine at the second position at codon 188 on one allele was observed in persons B–F (Figure 1C). The resulting amino acid exchange was threonine to lysine (AAG) (T188K). The mutation in patient C was clearly visible only after re-analysis with a different PCR-primer due to a polymorphism in the primer-binding region of the gene that is co-migrating with the silent mutation at codon 117. In fact, the patient C turned out to be heterozygous at three positions in the coding region of *PRNP*: the silent polymorphism at codon 117 and the codons 129 and 188. As the persons B, C, D, E were heterozygous (methionine (M)/V) at the polymorphic codon 129, the amino acid at this position in coupling with lysine at 188 was determined as M in all cases by molecular cloning. Person F was homozygous at codon 129 for M. The codon 117 polymorphism in patient C was coupled with V at codon 129. The mother of patient D (person G) did not carry a *PRNP* mutation.

### SNP screening

In order to test the prevalence of the T188R and the T188K mutations in sporadic CJD and in the healthy population a big study cohort consisting of 593 sCJD cases and 735 healthy individuals was screened. The mutations T188R and T188K or different nucleotide changes at codon 188 were not found in any of the examined persons.

### Histology/Immunohistochemistry *(Patient D)*

The histological examination of the tissue received from biopsy showed parts of the neocortex with spongiform changes and gliosis. Immunohistochemistry with an antibody against the human prion protein (PrP) (L42) revealed synaptic staining of the cortex.

**Figure 2:**
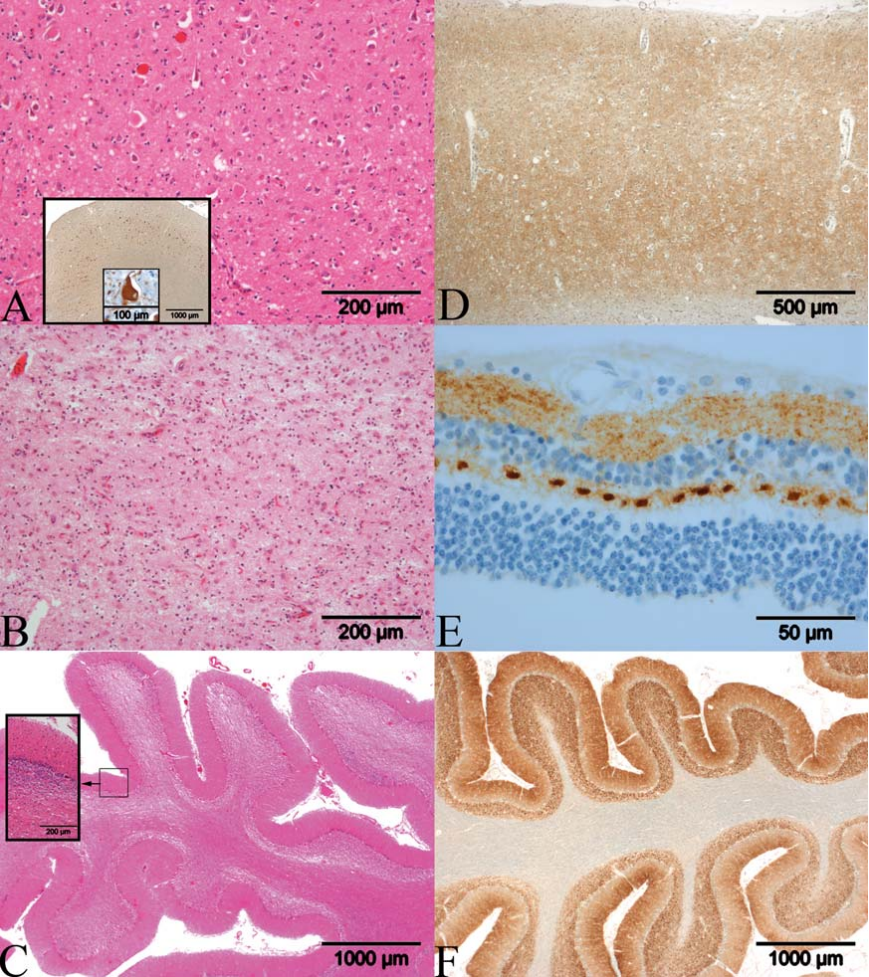
Post-mortem neuropathological findings in Patient D. A: Spongiform changes in the frontal cortex with numerous ballooned neurons (H&E, original magnification 100×); Inserts: Immunohistochemistry of ballooned neurons (αB-Crystalline; 20× and 200×). B: Status spongiosus in the occipital cortex (H&E, 100×). C: Cerebellar cortex with moderate spongiform changes of the molecular layer and severe degeneration of the granular cell layer (H&E, 20×). D–F: Immunohistochemistry with an antibody against PrP (L42) shows a synaptic pattern of PrP^Sc^ deposition. D: Frontal cortex (40×), E: Retina (400×). F: Cerebellar cortex (20×).

The neuropathological gross examination of the brain showed a moderate frontal atrophy, a diminution of cortical thickness in all regions, enlarged ventricles and a thin corpus callosum. The cerebellum revealed atrophy, especially of the superior lobe. The brainstem was macroscopically unaffected. The cerebral vessels showed mild arteriosclerotic changes. Histological examination of HE stained sections revealed severe spongiform changes in all layers of the cerebral cortex, astrocytic gliosis and neuronal depletion increasing from the frontal (Figure 2A) to the occipital lobe. Status spongiosus was found in the entorhinal, transentorhinal and occipital cortex (Figure 2B), putamen and caudate nucleus. The globus pallidus and cornu Ammonis showed only minor spongiform changes, which was more pronounced in the subiculum and amygdala. The medial and lateral part of the thalamus showed severe spongiform changes, astrocytic gliosis and neuronal loss. The nucleus basalis of Meynert revealed moderate gliosis. Furthermore numerous αB-crystalline immunoreactive ballooned neurons were found in the cortex of the cingulate gyrus, the frontal *(* Figure 2A insert) and temporal lobe and a small number in the external layer of entorhinal cortex. Mild demyelination and axonal loss were found mainly in the occipital lobe. The cerebellar cortex showed moderate spongiform changes of the molecular layer, severe degeneration of the granular cell layer with some torpedoes (Figure 2C). The Purkinje cells were mainly preserved. Moderate atrophy of cerebellar white matter was noted. The brainstem was nearly free of spongiform change, but astrocytic gliosis was found mainly in pons and the tegmentum of the whole brainstem. Immunohistochemistry with an antibody against the human prion protein (PrP) (L42) revealed strong synaptic staining of all layers of the cerebral and cerebellar cortex (Figure 2D,F), the hippocampus, the amygdala, the basal ganglia, the thalamus, the dentate nucleus, grey matter of the brainstem and the plexiform layers of the retina (Figure 2E). The PET blot of the hippocampus and cerebellar cortex showed intensive synaptic staining. The immunostaining with an antibody against hyperphosphorylated tau protein (AT8) revealed a few neurofibrillary tangles and neuropil threads in the transentorhinal cortex, the external and internal layer of the entorhinal cortex and few in the CA1 region and amygdala. These were not detectable using the Gallyas silver stain.

### Western blot analysis (*Patient D)*

**Figure 3:**
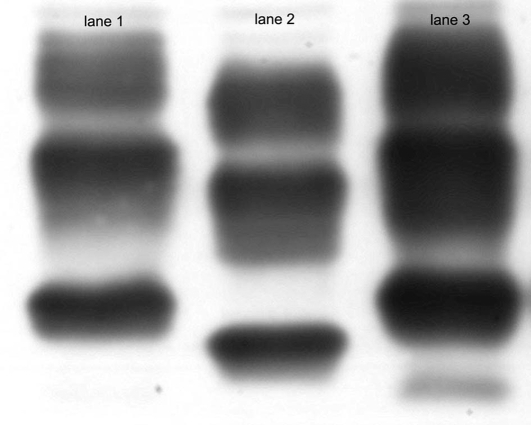
Western blot analysis of PrP after proteinase K digestion (lanes 1–3) using mAb 3F4. *Lane1:* PrP^Sc^ type 1 from a sporadic CJD case. *Lane 2*: PrP^Sc^ type 2 from a sporadic CJD case. *Lane 3*: Frontal cortex of patient D. As seen in lane 3 there is an additional band migrating at an apparent MW of 17 kDa.

The protease resistant PrP in brain homogenate from patient D was further analysed using Western blotting with the antibody 3F4. A PrP^Sc^ pattern type 1 was found (Figure 3, lane 3) similar to the type 1 pattern of sCJD case (MM1, Figure 3, lane 1). In addition there was a fragment migrating at about 17 kDa detected with the antibody 3F4. Additional bands have been reported in genetic prion diseases (mainly GSS), some also in sCJD and iCJD [19]–[22].

## Discussion

Here we present four patients carrying two different mutations at codon 188 of *PRNP* - T188K and T188R (for a summary of findings see Table 1). We show immunohistochemical data confirming that the T188K mutation is associated with a human prion disease (CJD) in one case. Patients B, C and D are from the same circumscribed region in Germany and were born not more than 140 km from each other; no familial relationship is known. Persons E and F, the sister and father of Patient D, have a T188K mutation and are healthy at ages 51 and 79 years respectively. The case with the T188R (Patient A) mutation is the only one reported to date [5], [23]. A T188K mutation of *PRNP* was published in an Austrian patient with rapid progressive dementia [24]. There is no histological description of these two cases. A T188A mutation was described in an Australian patient [25].

**Table 1:** Features of the prion diseases associated with mutations at codon 188. Abbreviations: AA = amino acid; n.a. = not available, IHC = Immunohistochemistry; m. = month; M = methionine; V = valine; y. = years.

	Patient A	Patient B	Patient C	Patient D (Patient)	Person E (Sister of D)	Person F (Father of D)	Patient – Austria [24]	Patient – Australia [25]
Mutation	T188R	T188K	T188K	T188K	T188K	T188K	T188K	T188A
Polymorphism at Codon 129/ AA on mutated allele	VV V	MV M	MV M	MV M	MV M	MM M	n.a.	MM M
Further polymorphism			A117A_129V					
Family history for neuro -degenerative diseases	negative	positive	negative	negative	negative	negative	negative	negative
Sex	F	F	F	F	F	M	n.a.	F
Age at onset (y.)	66	76	69	57	Healthy (at age 51 y.)	Healthy (at age 79 y.)	59	82
Duration of disease (m.)	16	5	7	13	not applicable	not applicable	(<1y.)	4
Progressive dementia	+	+	+	+			+	+
Myoclonus	-	+	+	+				+
Visual or cerebellar disturbance	+	+	+	+				+
Pyram./extra-pyram. dysfunction	-	+	+	+				+
Akinetic mutism	-	-	+	+				-
Symptoms at onset	visual impairment	ataxia	visual impairment, personality changes	aphasia, apraxia				memory deficits
Cerebral MRI	two unspecific symmetrical lesions in the frontal white matter	atrophy of the fronto-parietal cortex, little unspecific white matter lesions fronto-parietal, abnormal T2 signal in the basal ganglia.	mild enlargement of the ventricular spaces, periventricular unspecific white matter lesions in the centrum semiovale (T2)	cortical signal enhancing and diffusion abnormalities but no enhancing of basal ganglia			n.a.	minor cortical atrophy
PSWCs in EEG	+	+	+	+			n.a.	+
14-3-3	+	+	+	+			n.a.	+
Clinical diagnose according to [18]	criteria for CJD not fulfilled	Probable CJD	probable CJD	probable CJD	asymptomatic	asymptomatic	n.a.	Probable CJD
Neuropathology	n.a.	n.a.	n.a.	CJD	n.a.	n.a.	n.a.	CJD IHC negative

A comparison of the four cases with the T188K mutation (patients B–D of this study and the Austrian patient) shows a heterogeneous clinical picture concerning age at onset (57–76 y), disease duration (5–13 m), symptoms at onset (ataxia, personality change and aphasia) and MRI findings (Table1).

The cases B, C and D were originally classified as “probable sCJD” according to the diagnostic criteria for sCJD established by the WHO-guidelines as they showed the obligatory criteria and two (patient B) or all four (patients C and D) of the following clinical features: myoclonus, visual or cerebellar disturbances, pyramidal or extrapyramidal disturbances or akinetic mutism (Table 1) [18], [26]. In patient D a diagnosis of “definite CJD” was made after death. The genetic analysis revealed a mutation at codon 188 of *PRNP* (T188K) and heterozygosity at codon 129 of *PRNP* in the three cases. In patient D the PrP^Sc^ type 1 was found in the Western blot analysis (Parchi et al.1999) and the histological picture was indistinguishable from sCJD-MM1/MV1 [27], except for the numerous ballooned neurons in the cortex, which are not seen in sCJD-MM1.

A number of *PRNP* mutations are associated with characteristic cerebral MRI findings. A signal hyperintensity in the cerebral cortex more than in the basal ganglia (BG) was recognized as a characteristic feature in genetic CJD with the *PRNP* codon V180I mutation [28]. Mutations in codon 183 and 210 show abnormalities in the BG and cerebral cortex similar to sCJD [29]. Recent studies revealed that particular sCJD subtypes (MV2, VV1) also show specific characteristics on MRI [30], [31]. In the three cases with the T188K mutation no characteristic cerebral MRI pattern was identified. The typical T2 signal in the BG as proposed for sCJD criteria was found in patient B only [32]. In addition a hypoperfusion of the parietal cortex bilaterally was found in SPECT, but not on T2 weighted MRI 2–4 months after onset. The MRI findings in patient C were uncharacteristic. The isolated cortical hyperintensity on MRI and the missing BG sign in patient D may be due to the early time point when the single MRI was performed (one month after onset) [26], [33], [34].

Absence of a positive family history is noted in a significant proportion of genetic CJD cases (5–88%, depending on the mutation) and has drawn the attention to the issue of penetrance of mutations [4], [12]. Our findings suggest that the T188K mutation may not be fully penetrant as controversially discussed for other mutations (E200K, V210I) causing clinical phenotypes very similar to sCJD or that the pathogenic effect depends on additional factors [35]–[38]. Such factors could be the codon 129 polymorphism; whether this is of any significance in Person F, who is clinically not affected at age 79 and homozygous for M, is unknown at present.

The novel mutation at codon 188 in patient A (T188R) was found by routine sequencing of CJD suspects in the German surveillance [5], [23]. However, due to the negative family history and the absence of a genetic analysis of family members there is no evidence for the disease-causing effect of this mutation. No autopsy was performed. During clinical examinations CJD was strongly suspected because of the CJD-specific findings in EEG and CSF. The pathological VEP is common in CJD, even in patients without clinically affected visual system [39]. The MRI pattern was not typical as proposed for the CJD criteria because of the missing hyperintensity of the BG, even 12 months after onset, similar to three of four T188K mutations. Clinically patient A is similar to sporadic MM1/MV1 cases, because of the drastic visual problems. However, the disease duration (16 m.) exceeds the mean duration for this group (4–5 months). Homozygosity for V at codon 129 in this patient is remarkable since visual symptoms are very unusual in (sporadic) VV1 and VV2 patients. Dementia and ataxia in the course of the disease are common, ataxia is more typical of VV2. The PSWCs in EEG and the age at onset are similar to the sporadic VV2 subgroup, whereas the disease duration is at the outer limit for VV2.

Similar to all novel point mutations whose causative nature is not supported by a genetic linkage study, the question arises whether mutation T188K is truly pathogenic and not merely a polymorphism accidentally occurring in cases of sCJD. However, there are several arguments strongly supporting the pathogenic role of this mutation: (1) It was not identified in 735 healthy controls. (2) Threonine at codon 188 of *PRNP* is highly conserved throughout all mammalians, indicating that this mutation is likely to have a dramatic effect on the function of the prion protein [40], [41]. Codon 188 is situated in the C-terminal half of the second alpha-helix, a firmly structured part of the protein, and a drastic exchange like the substitution of threonine for a highly basic amino acid such as arginine (T188R) or lysine (T188K) would result in a structural destabilization. (3) Cell culture experiments are strongly suggestive of a pathogenic role for this mutation, since increased proteinase K (PK) resistance and insolubility in non-denaturing detergents have been observed [42].

The data presented here argue in favor of T188K being a pathogenic mutation causing genetic CJD. Since one individual with this mutation is now 79 years old and shows no signs of disease, this mutation may be associated with a penetrance under 100%. Further observations will have to show whether T188R is a pathogenic mutation.
