# Correction: Evidence for a Pathogenic Role of Different Mutations at Codon 188 of *PRNP*

**DOI:** 10.1371/annotation/3189bbdc-e736-4cb5-b1f2-9a5fb49536c6

**Published:** 2008-05-19

**Authors:** Sigrun Roeber, Eva-Maria Grasbon-Frodl, Otto Windl, Bjarne Krebs, Wei Xiang, Caren Vollmert, Thomas Illig, Andreas Schröter, Thomas Arzberger, Petra Weber, Inga Zerr, Hans A. Kretzschmar

There was an error in the author affiliations. The third author, Otto Windl, should be listed as an equally contributing author along with the first and second authors, Sigrun Roeber and Eva-Maria Grasbon-Frodl.

